# Molecular asymmetry in the 8-cell stage *Xenopus tropicalis* embryo described by single blastomere transcript sequencing

**DOI:** 10.1016/j.ydbio.2015.06.010

**Published:** 2015-12-15

**Authors:** Elena De Domenico, Nick D.L. Owens, Ian M. Grant, Rosa Gomes-Faria, Michael J. Gilchrist

**Affiliations:** a The Francis Crick Institute, Mill Hill Laboratory, The Ridgeway, Mill Hill, London NW7 1AA, UK

**Keywords:** Molecular asymmetry, Cortical rotation, Dorsal determinants, Spatial distribution of maternal mRNAs, Polar enrichment of mRNAs, *Xenopus tropicalis*

## Abstract

Correct development of the vertebrate body plan requires the early definition of two asymmetric, perpendicular axes. The first axis is established during oocyte maturation, and the second is established by symmetry breaking shortly after fertilization. The physical processes generating the second asymmetric, or dorsal–ventral, axis are well understood, but the specific molecular determinants, presumed to be maternal gene products, are poorly characterized. Whilst enrichment of maternal mRNAs at the animal and vegetal poles in both the oocyte and the early embryo has been studied, little is known about the distribution of maternal mRNAs along either the dorsal–ventral or left–right axes during the early cleavage stages. Here we report an unbiased analysis of the distribution of maternal mRNA on all axes of the *Xenopus tropicalis* 8-cell stage embryo, based on sequencing of single blastomeres whose positions within the embryo are known. Analysis of pooled data from complete sets of blastomeres from four embryos has identified 908 mRNAs enriched in either the animal or vegetal blastomeres, of which 793 are not previously reported as enriched. In contrast, we find no evidence for asymmetric distribution along either the dorsal–ventral or left–right axes. We confirm that animal pole enrichment is on average distinctly lower than vegetal pole enrichment, and that considerable variation is found between reported enrichment levels in different studies. We use publicly available data to show that there is a significant association between genes with human disease annotation and enrichment at the animal pole. Mutations in the human ortholog of the most animally enriched novel gene, *Slc35d1*, are causative for *Schneckenbecken dysplasia*, and we show that a similar phenotype is produced by depletion of the orthologous protein in *Xenopus* embryos.

## Highlights

• Analysis of spatial distribution of maternal mRNAs in *X*.*tropicalis* 8-cell embryos.
• Whole transcriptome sequencing of single blastomeres.
• 793 novel mRNAs found enriched in either the animal or vegetal blastomeres.
• No detectable asymmetry of mRNAs along either dorsal–ventral or left–right axes.
• Significant association between human disease orthologs and animal pole enrichment.

## 1 Introduction

The first steps of vertebrate development are guided by maternal mRNAs and proteins deposited during oogenesis. A subset of these maternal factors is likely to control asymmetry for correct axial development, and polymorphisms or deficiencies in these may lead to defects in body patterning. An understanding of which of these genes are asymmetrically expressed in the oocyte and early embryo, and also implicated in human disease, may help to direct research more effectively into the genetic causes of disease. *Xenopus* is an ideal model system both for the discovery of asymmetric and early acting genes, and the investigation of their role in development.

Bilaterian animals require the early definition of two asymmetric, perpendicular axes for correct development. The process of axial differentiation in the embryo is controlled by two main mechanisms: the action of maternal determinants transmitted to specific blastomeres, and cellular interactions mediated by various signaling molecules (Koga et al., 2012). Understanding the specific molecular determinants of these processes remains an important question in developmental biology. In many organisms, these early events are accomplished by the localization or sequestration of maternally synthesized proteins and mRNA (Danilchik et al., 2006). Mechanisms vary in non-vertebrate species (Gonczy and Rose, 2005, Kugler and Lasko, 2009, Steinhauer and Kalderon, 2006), but in vertebrates, the animal–vegetal axis is set up during oocyte maturation, and the dorsal–ventral axis is established at or shortly after fertilization (Croce and McClay, 2006).

**Fig. 1:**
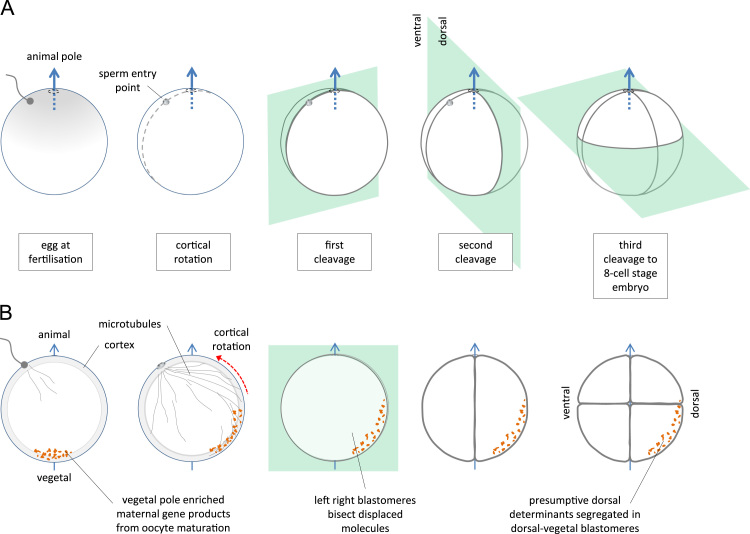
Cortical rotation and the segregation of maternal gene products in the cleavage stage embryo. A. Orientation of early cell divisions. The first cleavage plane is determined by the sperm entry point (SEP) and the animal–vegetal (A–V) axis. The second cleavage is orthogonal to the first but still contains the A–V axis; dorsal blastomeres are defined in the hemisphere opposite the SEP. The third cleavage is slightly above the equator. B. Displacement of maternal gene products. The egg contains vegetally localized maternal gene products. A microtubule network is set up at fertilization and the outer layer, or cortex, of the single cell embryo rotates to displace the vegetal cortical region away the SEP. Maternal gene products are displaced by movement with the cortex or by vesicle trafficking into the presumptive dorsal hemisphere, and are further segregated by subsequent cell divisions.

In *Xenopus*, the oocyte develops with radial symmetry around the animal–vegetal axis between the darkly pigmented animal pole and the lightly pigmented vegetal pole (Kageura, 1997). Fertilization can only take place in the animal hemisphere, which ensures that axial symmetry is broken in a stereotypical manner. Sperm entry initiates processes that reorganize the molecular content of the embryo, and the position of the sperm entry point defines the orientation of the other axes with reference to the animal–vegetal axis (Fig. 1A). Partway through the first cell cycle, the outer cortex of the embryo is rotated 30–40° degrees relative to the core cytoplasm; the vegetal polar region moving away from the sperm entry point (Gerhart et al., 1989). This *cortical rotation*, either directly or indirectly, transports molecules from the poles of the animal–vegetal axis to more equatorial regions (Houston, 2012) (Fig. 1B), which locates the dorsal end of the dorsal–ventral axis in the opposite hemisphere to the sperm entry point (Danilchik et al., 2006).

The cleavage plane of the first cell division is defined by the plane of cortical rotation. The second cleavage plane, orthogonal to the first but still containing the animal–vegetal axis, segregates the more dorsally distributed molecules from the more ventral ones. The third cleavage plane, slightly above the equator towards the animal pole, further compartmentalizes the segregated molecules (Denegre et al., 1998) (Fig. 1B). On the dorsal side, this segregation forms the basis for the Nieuwkoop center in the region of the vegetal–dorsal blastomeres, and later the Spemann organizer (De Robertis and Kuroda, 2004, Houston, 2012). In this scheme, the first cleavage determines the left and right halves of the animal, and while these exhibit superficial mirror symmetry, substantial left–right internal asymmetry develops over time. Although this has been investigated for some years, the molecular origins and timing of the development of left–right asymmetry are not well understood (reviewed in Blum et al., 2014; Coutelis et al., 2014).

Blastomere isolation and removal experiments have been carried out with *Xenopus* embryos. These have shown that separated halves of 2-cell stage embryos, and 8-cell stage embryos containing some combination of ventral and dorsal blastomeres, can still reach tailbud stage, whilst purely ventral halves give rise to cell masses lacking head or axial structure (Kageura and Yamana, 1983, Kageura and Yamana, 1984). Furthermore, the ability of transplants from oocytes or early embryos to induce the formation of a secondary axis in later stage embryos is evidence that some cytoplasmic dorsal determinants are localized early in specific regions of the embryos (Fujisue et al., 1993, Gallagher et al., 1991, Hainski and Moody, 1992, Kageura and Yamana, 1986).

The nature and identities of these dorsal determinants are not currently well defined. Previous experiments have shown that these putative cytoplasmic factors may be mRNA or proteins (Klein and King, 1988, Miyata et al., 1987, Shiokawa et al., 1984). Further, it has been argued that cytoplasmic polyadenylation may be the mechanism for the change in dorsal inducing activity of total RNA isolated from the dorsal lineage between the 8- and 16-cell stages (Pandur et al., 2002). Differential polyadenylation along the dorsal–ventral axis has been proposed to account for an observed dorsal enrichment of Wnt11 protein (Schroeder et al., 1999), and observed vesicle trafficking has been proposed to account for dorsal enrichment of Disheveled protein (Miller et al., 1999).

The process of mRNA segregation in the maturing oocyte has been well studied (King et al., 2005, Kloc et al., 2001, Kloc et al., 2002, Melton, 1987), and numbers of mRNAs with an asymmetric distribution on the animal–vegetal axis are published (Cuykendall and Houston, 2010, Houston, 2013, King et al., 2005).

Asymmetric distribution of maternal mRNAs in the early embryo has recently been investigated through transcriptional profiling of early cleavage stage embryos dissected in sections along the animal–vegetal axis (Grant et al., 2014). A qPCR study on 41 selected genes, analyzed individual blastomeres (identified as animal or vegetal) from 8-, 16- and 32-cell *Xenopus laevis* embryos (Flachsova et al., 2013) and inferred a lack of dorsal–ventral or left–right asymmetry from the failure of a principle components analysis to identify subgroups beyond animal and vegetal. There has, however, to the best of our knowledge, been no systematic, large-scale study of mRNA distribution over multiple axes in the blastula stage embryo. To comprehensively address the question of asymmetry of maternal mRNAs in the cleavage stage embryo, we have carefully disassembled 8-cell stage *Xenopus tropicalis* embryos, recording the position of each blastomere within the embryo, and performed whole transcriptome sequencing on the individual blastomeres.

## 2 Materials and methods

### 2.1 In vitro fertilization and blastomeres collection

Adult *X. tropicalis* females were induced to ovulate and eggs were fertilized, dejellied and cultured as previously described (Khokha et al., 2002). At the two-cell stage, embryos, in which the first cleavage furrow bisects equally the pigmentation on the animal pole, were selected as described (Grant et al., 2013). In this way the lighter area can be used as a marker of the future dorsal side (Klein, 1987, Masho, 1990). The selected embryos were transferred to cell dissociation buffer enzyme-free, pbs (Gibco, Life technologies), until the collection of the blastomeres at the 8-cell stage. At this stage, the selected embryos were manually devitellinated in dishes coated with 1% type V agarose. The blastomeres were separated and collected from embryos where the blastomeres were still linked by thin cytoplasmic bridges after devitellination, enabling us to record the positions of blastomeres within the embryo. Blastomeres were separated by creating a gentle flux of solution with the forceps against the cytoplasmic bridges still present. Full sets of undamaged blastomeres were collected in triReagent buffer (Sigma-Aldrich), immediately frozen in liquid nitrogen and stored at −80 °C.

### 2.2 Verification of dorsal–ventral axis orientation

Two-cell stage embryos were selected as described above, and the presumptive dorsal hemisphere was noted at the four cell stage. Dorsal and ventral blastomeres were injected with 1 nl of Alexa 488-conjugated dextran or Alexa 680-conjugated dextran (Thermo Fisher Scientific Inc.) respectively, and embryos were allowed to grow to tailbud or tadpole stage before being examined under fluorescence light microscopy to check orientation with respect to the original prediction of axis orientation.

### 2.3 RNA extraction and library preparation

RNA extraction from individual blastomeres was performed using RNeasy Micro Kit (Qiagen) with some modifications. The homogenization step was performed in triReagent buffer rather than RLT buffer and chloroform was added in a ratio of 1:5 for isolation of RNA. After centrifugation at 13,000 rpm for 30 min, the colorless upper aqueous phase was transferred into a new RNase free tube and manufacturer's instructions were followed. Samples were dissolved in 15 µl of nuclease-free water. RNA concentrations were measured with a Qubit 2.0 Fluorometer (Invitrogen), and RNA integrity was assessed using the Agilent 2100 bioanalyzer (Agilent Technologies, Santa Clara, CA). Only samples characterized by an RNA integrity number>8.0 were considered for library construction. For 4 embryos (7, 17, 24 and 30), rRNA-depleted RNA-seq libraries were prepared using the Ovation RNA-Seq System V2 (NuGEN Technologies Inc., San Carlos, CA). The libraries for Embryo 1 were prepared differently: cDNA was synthetized directly from the lysate cells using SMARTer® Ultra Low RNA Kit (Clonetech Laboratories, Inc., CA). All library protocols were combined with TruSeq DNA Sample Prep Kit v2 (Illumina, Inc.) and sequenced on the Illumina Hi-Seq 2500 platform to 60 bp, paired end.

### 2.4 Sequence data analysis

Paired end reads were aligned to a set of transcript sequences comprising the *X. tropicalis* v7.2 transcriptome and known off-genome sequences derived from EST assemblies, as described elsewhere (Collart et al., 2014). Alignment was performed using bowtie2 v2.1.0 (Langmead and Salzberg, 2012) with parameters “-k 200 -X 800 -t--rdg 6,5 --rfg 6,5 --score-min L,-.6,-.4--no-discordant --no-mixed”. Read pairs aligning uniquely with up to two mismatches per read were counted. We calculated the library size for each sample as total counted reads, excluding counts to ribosomal and other non-gene model sequences. To report read counts we normalized by the library size to a standardized library of 25 million reads. We perform differential expression tests on raw counts using the library size as a covariate. Genes with at least 10 normalized read counts in at least four blastomeres of any one embryo were considered for differential expression analysis.

To establish the set of genes differentially expressed over any of the animal–vegetal, dorsal–ventral or left–right axes, we combine negative binomial regression with a likelihood ratio test. We perform identical tests for single and pooled embryos:

Let $s_{j}$ be the library size for sample j, and let $r_{ij}$ be the raw count of gene i in sample j. We assume that $r_{ij}$ is negative binomially distributed (Cameron and Trivedi, 1998) with mean $\mu_{ij}$ and variance $\mu_{ij}+\;\alpha_{i}\mu_{ij}^{2}$, with $\alpha_{i}$ the dispersion parameter for gene i. We consider the null hypothesis that a gene is equally expressed in all blastomeres: $\log\;\mu_{ij}=\;\log\;s_{j}+\beta_{i,null}$, and an alternative hypothesis for each axis that the gene is differentially expressed over axis a: $\log\;\mu_{ij}=\;\log\;s_{j}+a_{j}\beta_{i,a}+\;{\bar{a}}_{j}\beta_{i,\bar{a}}$, where $a_{j}$ and ${\bar{a}}_{j}$ are indicator variables determining which side of the axis a the blastomere lies, and the β parameters are log mean expression levels. For each gene, we compute maximum likelihood estimates of the $\alpha_{i}$ and $\beta_{i,*}$ parameters under the null and alternative hypotheses. We perform a likelihood ratio test: if $\ell_{N}$ and $\ell_{A}$ are the log-likelihoods of the null and alternative models respectively then the test statistic $\;-2\left(\ell_{N}-\ell_{A}\right)\;$is assumed to be $\chi_{1}^{2}$ distributed under the null hypothesis. We control the false discovery rate (FDR) using the Benjamini–Hochberg method (Benjamini and Hochberg, 1995), and determine the genes differentially expressed with an FDR<0.05. We also consider a different form for the alternative in which we simultaneously test for differential segregation over the combinations of two axes or all three axes: $\log\;\mu_{ij}=\;\log\;s_{j}+\sum_{q}a_{q,j}\beta_{i,a_{q}}+{\bar{a}}_{q,j}\beta_{i{\bar{a}}_{q}}$, with $a_{q}$ indicating the relevant axes.

### 2.5 Annotating genes from Affymetrix microarray probe-set IDs

To improve the identification of genes associated with the *X. laevis* Affymetrix probe-set IDs, and in particular to link Affymetrix IDs to our *X. tropicalis* reference transcript set, we combined BLAST search data with paired-end information linking assembled EST clusters. We used the following sets of sequences: Affymetrix target sequences for v1 and v2 microarray probes sets; EST and cDNA sequences for accession numbers associated with Affymetrix probes sets; assembled EST consensus sequences from builds Xt7 (*X. tropicalis*) and Xl4 (*X. laevis*) at http://genomics.nimr.mrc.ac.uk/apps/ESTs/ (Gilchrist et al., 2004); and our transcript reference set, which is reported previously (Collart et al., 2014). Affymetrix sequence files were BLASTed against all other files, the assembled EST cluster consensus sequences were BLASTed against the transcript reference sequences, with an *e*-value limit of 10^−20^, and best hits per query sequence with a percent identity of at least 85% were retained. Alternative routes through the data for each Affymetrix ID to transcript reference IDs were computed: (i) from Affymetrix ID direct to transcript reference IDs, (ii) indirectly via either set of EST cluster consensus sequences, and (iii) indirectly via two EST clusters linked by paired end information. Identifications were accepted where a clear majority of computed links arrived at the same target; identification was noted as ambiguous where there was no clear best target; Affymetrix IDs were annotated as unidentified if there were no links to reference transcript sequences. Paired Affymetrix and reference transcript identifiers are available in Supplementary file 1.

### 2.6 Finding orthologous human disease genes

To identify *X. tropicalis* orthologs of human disease genes we first downloaded human disease gene data from http://www.cbs.dtu.dk/suppl/dgf/disease_complexes/networks/complexes.zip (Lage et al., 2008). Diseases associated with human protein complexes were transferred to all the genes within each complex. Orthology relationships between human and *X. tropicalis* genes were established via Ensembl and NCBI Entrez gene IDs and matching to records on Xenbase gene pages (James-Zorn et al., 2013). Human disease annotation was also taken directly from Xenbase gene records (James-Zorn et al., 2013).

### 2.7 Morpholino knockdown analysis of Slc35d1

An antisense morpholino oligonucleotide, sequence 5′-ACGTCTACCGACTTCCGCCATG-3′, was designed to target the 5′ untranslated region of the *X. tropicalis* gene solute carrier family 35, member D1 (S*lc35d1*) and block translation. A standard control morpholino was also used to check the toxicity of the concentrations used. All morpholinos were supplied by GeneTools (Philomath, OR). Doses of 10, 20, 30 and 40 ng of Slc35d1-MO or control-MO were injected into the single-celled zygotes with fluorescein-labeled dextran. After injection, embryos were raised to stage 33 and fixed in MEMFA solution. Embryos were stained for cartilage with Alcian Blue using standard procedures (Klymkowsky and Hanken, 1991).

Slc35d1-HA tagged mRNA was *in vitro* synthetized and injected in one-cell stage embryos to check morpholino effectiveness (Fig. 5A). The pCMV-slc35d1-HA was linearized with ApaI and transcribed with the mMessage mMachine SP6 kit (Ambion). A total of 200 pg of mRNA was injected into the one cell embryos both alone and in conjunction with the Slc35d1-MO. Embryos were collected 5 h after injection (stage 8/9) for protein extraction and western blot analysis.

Collected embryos were homogenized in lysis buffer (1 M Tris pH7.5, 5 M NaCl, 0.5 M EGTA, 10% Igepal CA-630, and 0,25% Sodium deoxycholate) containing protease inhibitor mixture (Roche) and centrifuged at top speed at 4 °C. Cell lysates were incubated with polyclonal ChIP Grade anti-HA tag antibody (Abcam). Immunocomplexes were precipitated using Dynabeads protein A (Invitrogen) and separated by SDS-PAGE (12%). After electrophoresis, the proteins were transferred to a PVDF membrane 0.45 mm (BioRad). After blocking the membrane in Odissey blocking buffer (LI-COR Bioscience, Ltd.-UK) in TBS, the blot was incubated overnight at 4 °C with the antibody. The blot was then washed and incubated with IRDye 800 CW goat-anti-mouse IgG (LI-COR Bioscience Ltd., UK) and imaged using the LiCOR Odyssey system.

## 3 Results and discussion

### 3.1 Asymmetrically distributed mRNAs

**Fig. 2:**
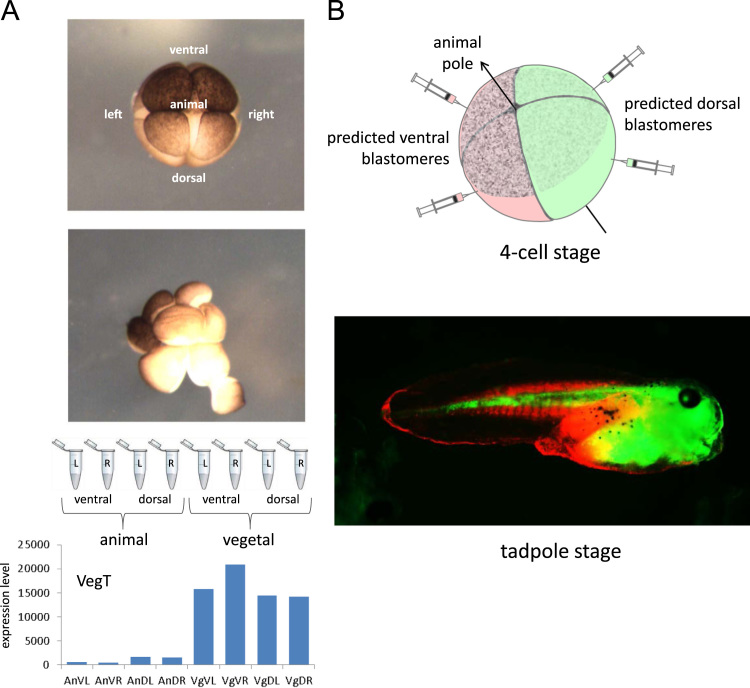
A. Disassembling and sequencing the 8-cell stage embryo. Photograph of the intact 8-cell embryo, and during microsurgical disassembly; note lighter dorsal/animal blastomeres. Sequencing of single blastomeres from a single embryo, showing layout of displayed data for known vegetally enriched gene, *VegT*. B. Dye tracing experiment to confirm correct identification of dorsal–ventral axis at the 4-cell, and by extension at the 8-cell, stage. The dorsal/animal quadrant is identified by more lightly pigmented blastomeres.

To investigate the distribution of maternal mRNAs along the three principle axes of the embryo, we carefully disassembled 8-cell stage *X. tropicalis* embryos showing stereotypical orientation of the first cleavage plane (Grant et al., 2013), noting the axial location of each blastomere (vegetal–dorsal–left, etc.) (Fig. 2A). In total we analyzed five such embryos, each from a different clutch, constructing RNA-seq libraries for complete sets of individual blastomeres from each embryo. For four of the embryos we made ribosomal RNA-depleted libraries using the NuGEN Ovation v2 kit to detect both unadenylated and polyadenylated mRNAs (total mRNA), and for the fifth embryo we used the Clonetech Laboratories low RNA SMARTer kit to detect polyadenylated mRNA (polyA+ mRNA). RNA-seq data sets were labeled for embryo and blastomere position, mapped to the *X. tropicalis* transcriptome and normalized. The normalized gene counts for the blastomeres can be found in Supplementary file 2. See Section 2 for more details.

To determine whether the dorsal pole of the dorsal–ventral axis was correctly predicted at the 8-cell stage we performed fluorescent dye lineage tracing on 20 embryos from different clutches with injection at the 4-cell stage (Fig. 2B and Section 2). There were no inconsistencies: daughter cells from the predicted dorsal blastomeres from the 4-cell stage made up the head, anterior trunk and spinal cord in all cases (Fig. 2B).

We detected mRNA for 12,951 genes at a sufficient level to test for asymmetric distribution (Methods). We evaluated asymmetry in two ways: in the individual embryos and in pooled data from all embryos sequenced for total RNA (Ovation kits). The single embryo data enabled us to assess the variability of mRNA asymmetry between embryos, and allowed us to explore the possibility that we had misidentified the dorsal end of the dorsal–ventral axis in some embryos.

We used the same method for detecting asymmetry in both the single and pooled embryo data. In pooled tests we treated RNA-seq counts from the equivalently positioned blastomeres in different embryos as biological replicates. For each gene we performed likelihood ratio tests, comparing two hypotheses for each axis: first that the distribution of mRNA was identical in all blastomeres, second that the distribution of mRNAs was characterized by different means at either end of the axis. Resulting *p*-values were controlled with Benjamini–Hochberg FDR (Benjamini and Hochberg, 1995). See Section 2 for details. The pooled data provided greater statistical power to detect asymmetry for a given gene.

**Fig. 3:**
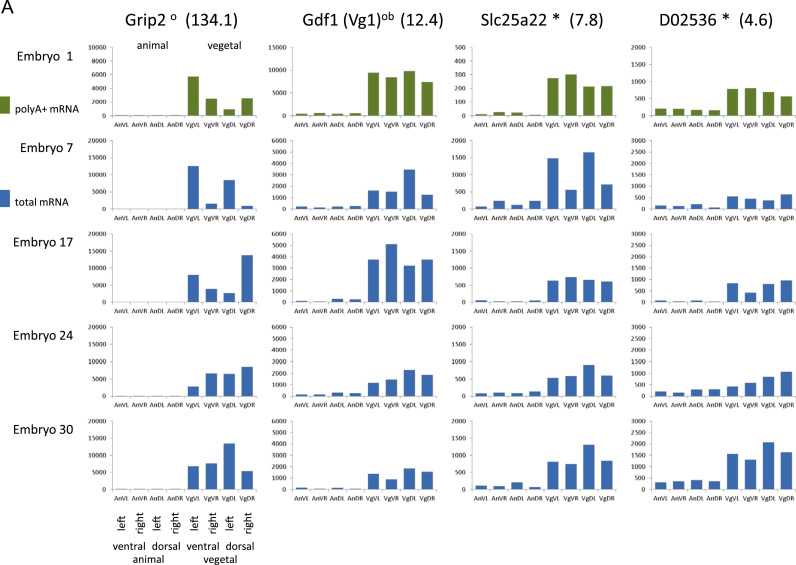

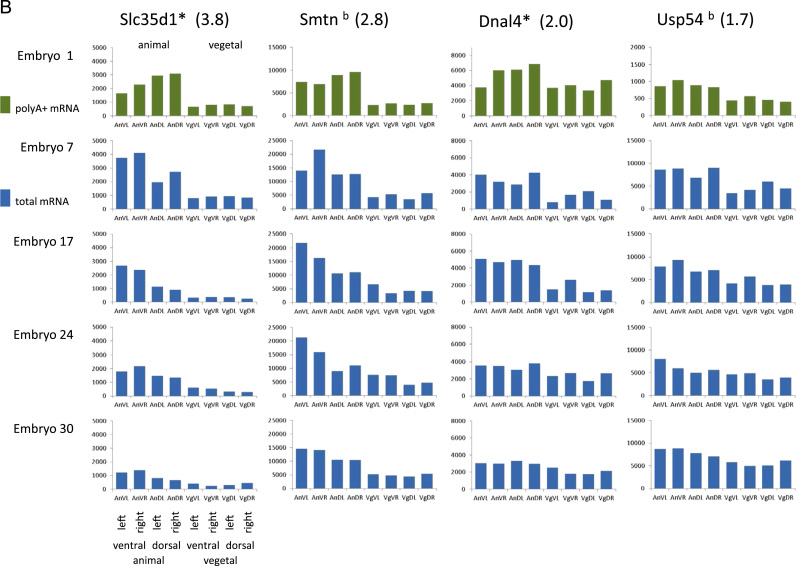
Examples of single blastomere sequencing data for 8-cell stage embryos. Each panel contains sequence data for five embryos. Green is from polyA+ SMARTer low-RNA libraries, blue are from total mRNA Ovation v2 libraries. Suffixes: *novel in this study, °previously found in oocyte data (Cuykendall and Houston, 2010), ^b^previously found in blastomeres data (Grant et al., 2014). Figures in brackets are fold-changes in this study. A. Vegetal pole enrichment. B. Animal pole enrichment.

**Fig. 4:**
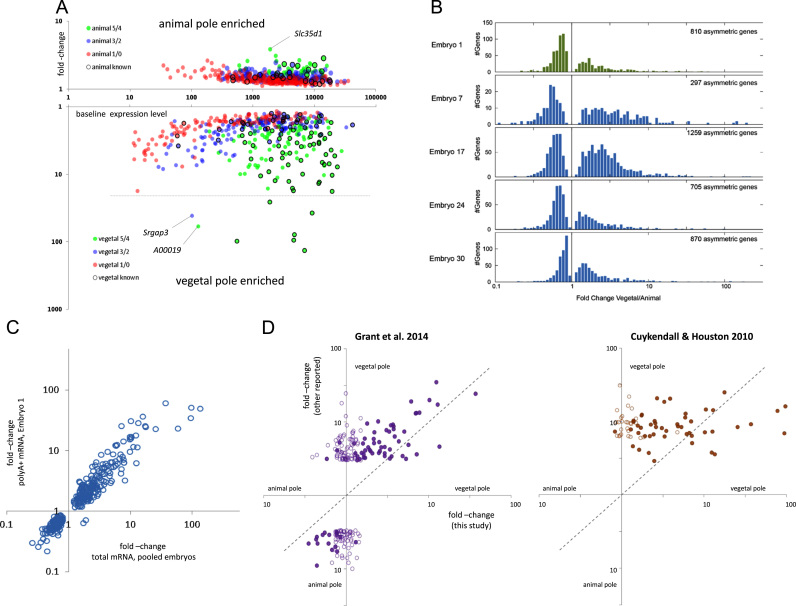
Variation in enrichment within and between studies. A. Variation of fold-change with expression level at animal and vegetal poles in this study. Scatter plot of mean expression level at the enriched pole against fold change measured between the poles for the 908 enriched genes in the pooled embryo analysis. Points are color coded according to the numbers of individual embryos in which significant asymmetric expression was detected in the single embryo analysis, see Key. Genes are clearly segregated according to the consistency of detection at the single embryo level. The much greater fold-change for vegetal pole enrichment is clearly visible (note the log scale). Genes enriched in our data and previously published are highlighted (black circles), and the most enriched novel mRNAs are indicated. B. Distribution of fold-changes at either pole for individual embryos. C**.** Agreement of measured fold-changes between the pooled total mRNA data and the polyA+ mRNA data of Embryo 1: Spearman correlation=0.94. D. Correlation of gene enrichment between this study and earlier work. Scatter plots of enrichment fold-change on the animal–vegetal axis for genes in this study previously reported as enriched in other studies. Left panel, blastomere data (Grant et al., 2014), right panel, oocyte data (Cuykendall and Houston, 2010). Full circles: genes enriched in present study with FDR<0.05; open circles: genes not found enriched, with FDR>0.05, fold-change in our data is simple ratio of vegetal/animal pole reads.

We first present data from the pooled analysis of blastomeres sequenced for total mRNA. We found 908 genes differentially expressed between animal and vegetal blastomeres with an FDR<0.05. We did not find any genes differentially expressed along either the dorsal–ventral or left–right axes. In addition, we tested for and found no genes differentially expressed over any two axes taken together. Of the genes differentially expressed over the animal–vegetal axis, we found 448 with increased expression in the vegetal hemisphere with a mean fold-change of 4.2 (ranging from 1.1 to 134.1), and 460 with increased expression in the animal hemisphere with a mean fold-change of 1.5 (ranging from 1.1 to 3.8). Examples of measured expression levels in the individual blastomeres for a range of genes are shown, for both vegetal pole enrichment (Fig. 3A) and animal pole enrichment (Fig. 3B). For both groups of asymmetrically distributed mRNAs there was little correlation between fold-change and expression level, whilst the difference between the levels of enrichment at the two poles was striking (Fig. 4A).

Analysis of the data at the single embryo level revealed that genes shown to be asymmetrically distributed in the pooled analysis were not necessarily found to be significantly differentially expressed in each individual embryo. Consistency was greater at both higher fold changes and baseline expression levels (Fig. 4A), and we note that a small proportion of genes, detected as asymmetrically distributed in the pooled data, were not detected as such in any embryo on its own (Table 1). For a given gene the measured fold change varied between embryos: summarizing over all genes found asymmetric at the pooled level, we found that ~93% of the fold changes found in individual embryos are within 2-fold of the fold change found in the pooled embryo tests. The variation in fold-change, and the differences between the poles, can be seen clearly in the distribution of fold-changes over all asymmetric genes for each individual embryo (Fig. 4B). The range of fold-change is always greater on the vegetal pole side, with roughly the same number of genes compressed into a narrower range on the animal side. We report the range of fold changes from individual embryos in the tabulated lists of differentially expressed genes (Table 2 and Supplementary file 3) as well as the number of individual embryos in which they were found significantly differentially expressed. That we do not find *significant* differential expression in all embryos at the single embryo level (for genes found asymmetric at the pooled level), is a consequence of the lack of statistical power at this level, and not the lack of asymmetry. In fact we find that, in 95% of those cases where the significance fell below our (FDR<0.05) threshold, the fold-change still agreed in direction with the fold-change from the pooled analysis.

We also used the single embryo data to verify that failure to detect asymmetry along the dorsal–ventral or left–right axes was not due to occasional mis-identification of the orientation of the dorsal–ventral axis in the embryos before disassembly. To do this, we looked for genes with opposing asymmetry along either the dorsal–ventral or left–right axes in data from different single embryos. We found no examples of genes showing such behavior, with FDR<0.05. We conclude that any dorsal–ventral or left–right asymmetries in these embryos would be below the levels at which we have detected asymmetry on the animal–vegetal axis (i.e.<1.12-fold, see Table 1), and therefore not at levels where we might expect the asymmetric distribution to have functional consequences.

We report here the 74 genes whose maternal mRNAs were consistently detected as differentially expressed over all five embryos (Table 2): 65 enriched in the vegetal hemisphere with fold-changes ranging from 134-fold to just under 2-fold, and 9 enriched in the animal hemisphere with fold-changes ranging from 3.8-fold to 1.7-fold. The full list of 908 differentially expressed genes is provided in Supplementary file 3.

To investigate the relative asymmetry of polyadenylated transcripts specifically, we compared the fold-change values from the pooled embryo data (total mRNA) on the animal–vegetal axis with those calculated for Embryo 1 (polyA+ mRNA). We found good agreement between the fold changes (Fig. 4C), with a Spearman correlation of 0.94. Furthermore, where there was more than one embryo with a significant fold-change in the single embryo total mRNA data, over half of the polyA+ fold-changes for Embryo 1 (133/262) were inside the range of these values, and including all genes where at least one embryo showed a significant fold-change, over 94% of the polyA+ fold-changes (405/430) were within 2-fold either way of the average of the significant fold-changes in the total mRNA data. In general, the polyadenylation status did not make a marked or systematic difference to the asymmetry detected along the animal–vegetal axis.

### 3.2 Comparison with previously published genes

We next compared our data with asymmetrically distributed maternal mRNAs reported in earlier studies. The majority of these data were from two microarray studies in *X. laevis*: manually dissected vegetal cortices from stage VI oocytes compared with whole oocytes (Cuykendall and Houston, 2010), and 8-cell embryos dissected in sections along the animal–vegetal axis comparing the animal blastomeres with a polar section of the vegetal blastomeres (Grant et al., 2014). The first of these provided a list of 120 genes enriched at least 6-fold in the vegetal cortex of the oocyte; the second, a list of 285 genes enriched at least 3-fold (108 animal pole and 177 vegetal pole) in the dissected embryos. In addition, 32 genes (14/18 animal/vegetal) from a qPCR study of individual blastomeres (Flachsova et al., 2013), 38 genes (30/8 animal/vegetal) from a review of oocyte mRNA asymmetry (King et al., 2005), and 12 genes from other publications (Betley et al., 2002, Birsoy et al., 2005, Colozza and De Robertis, 2014, Freeman et al., 2008, Horvay et al., 2006, Kloc and Chan, 2007, Machado et al., 2005, Nakaya et al., 2004, Quick and Serrano, 2005, Rutenberg et al., 2002, Tarbashevich et al., 2011, Tarbashevich et al., 2007).

For some of these *X. laevis* studies, the orthologous genes in our *X. tropicalis* data were identified manually, by comparison of gene symbols and known historical synonyms. However, the majority of genes were reported with Affymetrix microarray probe-set IDs, and these were often not well annotated. To identify them we used a computational approach to link the *X. laevis* Affymetrix IDs (v1 and v2) to the *X. tropicalis* gene identifiers used here (Section 2). This enabled us to link 28,768 out of 32,317 Affymetrix IDs (v2) to our reference sequence data, of which 2961 were ambiguous. We note that around 20% of our *X. tropicalis* genes were not associated with an *X. laevis* Affymetrix ID.

Collating genes between the different studies, we found that there were 342 mRNAs previously reported in the literature as exhibiting animal or vegetal enrichment in our reference transcript set. Surprisingly, we found only 115 of these in our list of 908 asymmetrically expressed genes from our pooled blastomere analysis, even though we detected differential expression at much smaller fold-changes than the thresholds used in the earlier studies. We report here the (74) mRNAs in our data for which we found significant asymmetry in all five embryos analyzed independently (Table 2). Even in this strongly asymmetric sub-set of genes we found less than half (35) had been previously identified in one or more earlier studies. The full list is provided in Supplementary file 3.

We next looked at the correlation between the reported fold-changes of the enriched genes in our study and those in the earlier microarray studies. Correlation with the animal/vegetal pole embryo data (Grant et al., 2014) appeared to be moderate, whilst there was little or no correlation with the vegetal cortex oocyte data (Cuykendall and Houston, 2010) (Fig. 4D). In addition, genes in our data with a slight difference in expression level along the animal–vegetal axis but with an FDR>0.05, were equally likely to have been previously reported enriched at the opposite pole (Fig. 4D). On average we reported lower fold changes than in previous studies, notably at the animal pole (Fig. 4D), where, for the set of genes in common, we report enrichment up to 3-fold compared to the 3- to 10-fold reported in the earlier study (Grant et al., 2014).

Inconsistent fold-changes between studies have been reported previously (Grant et al., 2013) and we suggest that this might not be unexpected, given both the difference in experimental designs, and the known natural variability of maternal mRNAs between clutches (Collart et al., 2014). The present study looks at differential expression between whole blastomeres and averages out variation within the blastomeres; the earlier embryo study compared the vegetal tips of the vegetal blastomeres with whole animal blastomeres; and the earlier oocyte study compared the polar vegetal cortex of the oocyte with whole oocytes. Without a more detailed understanding of the global distribution of mRNAs within the oocyte, egg and early blastomeres, it is difficult to understand the origin of the observed variation of fold changes between different studies.

### 3.3 Novel segregated mRNAs

In order to identify novel, asymmetrically distributed mRNAs in a manner consistent with previous studies we used the same 3-fold threshold as in the animal/vegetal pole embryo data (Grant et al., 2014). With this threshold we found 128 enriched mRNAs, of which 85 have not been previously described as enriched. All but two were enriched at the vegetal pole. We note that these represent a mix of transcripts with and without Affymetrix IDs (50/35 respectively), of which the former could have been detected in the earlier studies, whilst the latter would not.

Six of the top eight vegetally enriched mRNAs, measured by fold-change, were known (Fig. 4A), but the fifth and sixth most enriched were novel: an unidentified gene (Xetro.A00019), and *Srgap3* (slit-robo rho GTPase activating protein 3; Xetro.D02367) (Fig. 4A lower). The expression levels of both are in the bottom 15% of vegetally enriched genes (and the bottom 30% of all tested genes). *Srgap3* is a well conserved protein coding gene, although not detected in the assembled *X. tropicalis* EST data (Gilchrist et al., 2004). We find its expression to be highly variable within each embryo, but in this respect it is similar to *Grip2* (Fig. 3A), which has been shown to be important for the proper positioning of primordial germ cells (Tarbashevich et al., 2007). In contrast, *A00019* is difficult to characterize. It does not appear to be a protein coding gene; searching the NCBI nr protein database with BLASTx returns no hits.

The most enriched gene in the animal hemisphere, measured by fold-change, was novel: *Slc35d1*, solute carrier family 35 (UDP-glucuronic acid/UDP-N-acetylgalactosamine dual transporter), member D1 (Fig. 4A upper). *Slc35d1* is reported to be critical to chondroitin sulfate synthesis in cartilage and skeletal development in mouse and human (Hiraoka et al., 2007). There are many more novel, asymmetrically expressed genes at lower levels of enrichment, and the full list can be found in Supplementary file 3. Of our list of 908 asymmetrically expressed genes, 793 have not been previously reported; of the known ones, 88 were enriched at the vegetal pole and 27 at the animal pole.

### 3.4 Human disease genes

As *Xenopus* is an excellent model system for studying early development in vertebrates, we were interested to see which genes with asymmetrically distributed maternal mRNAs were orthologous to known human disease or disease associated genes.

To explore the association with disease we linked 4173 *X. tropicalis* genes (about 20% of the estimated total number of genes) through orthology data to human genes associated with disease in a high confidence protein interactome (Lage et al., 2008) covering 1048 human disease entries in OMIM (Methods). In addition we downloaded a set of 2653 *X. tropicalis* genes orthologous to human disease gene directly from the *Xenopus* community database, Xenbase (James-Zorn et al., 2013). Together these yielded a set of 5428 human disease orthologs which we used to test for enrichment of disease genes amongst the asymmetrically distributed cohorts. The full list of genes and diseases can be found in Supplementary file 4.

We first tested to see whether genes with asymmetric expression were enriched for human disease orthologs. Out of the 12,951 genes tested for differential expression, we found 4068 with a human disease annotation. Of the 908 asymmetric genes, 312 had a human disease annotation, compared to the 285 genes we would have expected by chance, suggesting a mild association between asymmetry and disease (*p*=0.049 Fisher Exact test). We then looked to see if the direction of the enrichment was related to the degree of association. Enriched at the vegetal pole, we found 135/448 genes with a disease annotation, slightly less than would be expected by chance. In contrast, amongst the genes enriched at the animal pole, we found 177/460 genes with a disease annotation, 32 more than would be expected by chance (*p*=0.001 Fisher Exact test). We conclude that there is a significant association between genes with human disease annotation and enrichment at the animal pole, and that the weak association between human disease and asymmetric genes *per se* is due to those genes found enriched at the animal pole. This was a somewhat counter-intuitive result, as we expected the significantly larger asymmetries to be more associated with disease. Human disease associations with genes that were consistently found asymmetrically expressed are reported within Table 2, and the remainder can be found in Supplementary file 3.

**Fig. 5:**
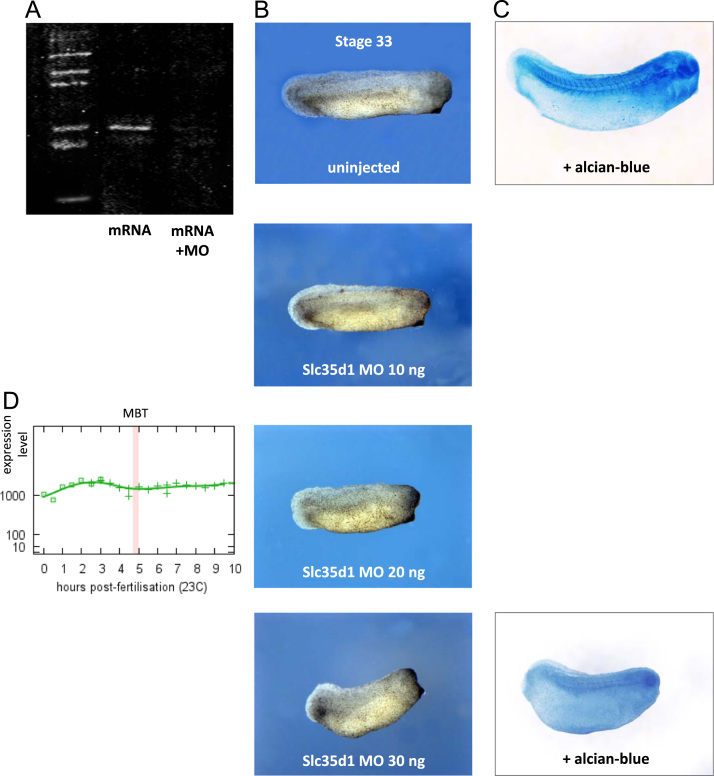
Exploration of morpholino knockdown of Slc35d1. A. In vitro validation of Slc35d1-MO blocking translation of HA tagged Slc35d1 protein. B**.** Body axis shortening phenotype of Slc35d1-MO injected embryos, with approximate dosage effect up to 30 ng injected MO, at stage 33. Penetrance of the phenotype was at least 94% over all concentrations (see Table 3). C**.** Comparison of uninjected and 30 ng Slc35d1-MO injected embryos under Alcian Blue staining suggests major loss of skeletal development. D**.** Expression profile of *Slc35d1* shows significant levels of polyA+ mRNA maintained into gastrulation (Collart et al., 2014).

The most asymmetrically expressed novel gene in the animal blastomeres, *Slc35d1*, is implicated in the human disease *Schneckenbecken dysplasia* (Borochowitz et al., 1986), considered to be associated with a dwarfism phenotype (Lahmar-Boufaroua et al., 2009). To explore the importance of this gene in *X. tropicalis* during early development we injected a translation blocking morpholino oligonucleotide at different concentrations (Section 2). Effectiveness of the Slc35d1-MO was determined separately by injection of Slc35d1-HA mRNA. Control embryos were injected with matched levels of standard control morpholino, and were developmentally unaffected until at least the tadpole stage, when observations ceased. Slc35d1-MO injected animals showed shorter dorsal axis with poor development of dorsal tissues and head, and by tadpole stage had died and degraded. Increasing concentrations of injected morpholino caused progressive increase in the severity of the morphant phenotype (Fig. 5B), and penetrance was high (at least 94%) at all concentrations (Table 3). To highlight cartilage tissue we stained embryos at tailbud stage with Alcian blue, as described previously (Klymkowsky and Hanken, 1991), which showed partial or complete loss of vertebrate cartilage primordia (Fig. 5C). It is unclear what the role of animal pole enrichment is for the function of this gene. We note that the expression level of the gene is maintained well into gastrulation (Fig. 5D), although whether the asymmetry is maintained during zygotic transcription is not known. Our findings suggest that *Xenopus* embryos develop a lethal form of skeletal dysplasia in a similar manner to Slc35d1-deficient mice (Hiraoka et al., 2007) and humans, highlighting the power of the *Xenopus* model system for the study of prenatal dysplasia, and potentially of other developmental disease syndromes.

In addition, amongst the more consistently asymmetric genes enriched at the animal pole (in at least 4/5 single embryo tests) we find four further novel genes with human disease associations and greater than two-fold enrichment. In decreasing order of enrichment they are: *Mprip*, 3.1-fold, implicated in ogna type epidermolysis bullosa simplex, muscular dystrophy, and cryptogenic cirrhosis; *Fam82a2*, 2.3-fold, implicated in Pelger–Huet anomaly, greenberg skeletal dysplasia, familial hypercholanemia, and other diseases; *Acaca*, 2.2-fold, implicated in acetyl-coa carboxylase deficiency; and *Dck.2*, 2.1-fold, implicated in partial adenosine deaminase deficiency, mitochondrial myopathy and sideroblastic anemia 1, and other diseases.

Amongst the more consistently asymmetric genes enriched at the vegetal pole (in at least 4/5 single embryo tests) we find six novel genes with human disease associations and greater than four-fold enrichment. In decreasing order of enrichment they are: *Farp2*, 16.2-fold, implicated in Ehlers–Danlos syndrome type viib, type iii and iv osteogenesis imperfect, and other diseases; *Slc25a22*, 7.8-fold, implicated in early infantile encephalopathy 3; *Pld2*, 7.5-fold, implicated in diabetes mellitus type 2, generalized congenital lipodystrophy type 1, lung cancer, and other diseases; *Mark4*, 4.7-fold, implicated in Hermansky–Pudlak syndrome 1; *D02536*, 4.6-fold, implicated in autosomal dominant robinow syndrome, and amongst others, early onset parkinson disease 6; and *Plk3*, 4.5-fold, implicated in esophageal squamous cell carcinoma, lung cancer, and other diseases.

Although it is not clear how many of these disease associations will ultimately be traceable to asymmetries in the early embryo, these data underline the viability of *X. tropicalis* as a model for human disease, especially where the development of the early embryo is affected. The rapidly developing and accessible early embryos enable the simple observation of the onset of the disease phenotype, and would provide a large scale and cost effective vehicle for screening potential therapeutic interventions.

### 3.5 Insights into the behavior of candidate dorsalizing proteins

**Fig. 6:**
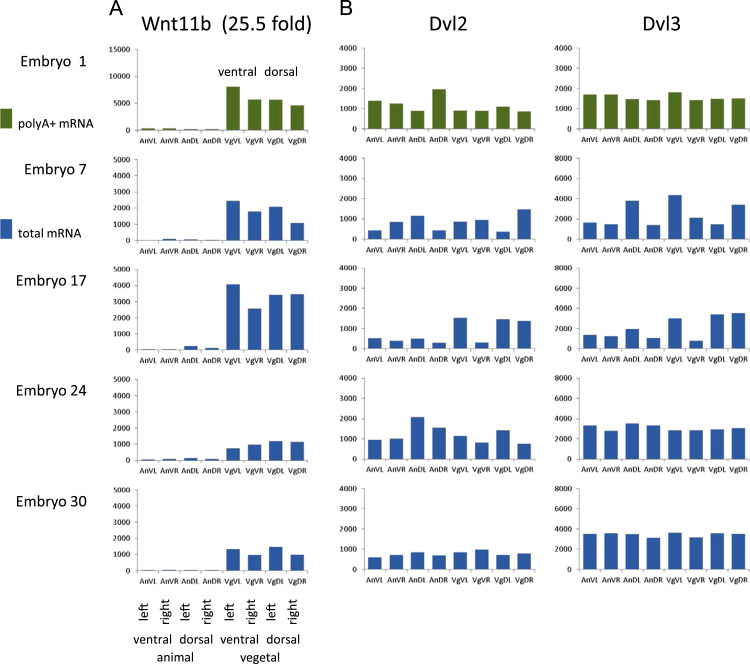
Distribution of gene expression for candidate dorsalizing factors in the 8-cell embryo. A. Expression of maternal *Wnt11b* shows substantial animal–vegetal asymmetry, without any consistent dorsal–ventral segregation, and, specifically, little difference between dorsal and ventral blastomeres, in either the PolyA+ or total mRNA prepared libraries. B. Expression of maternal *Disheveled* is relatively uniform throughout the early cleavage stage embryo, suggesting that these mRNAs were not concentrated at the vegetal pole of the oocyte or the fertilized egg.

It has been proposed that differential polyadenylation, observed along the dorsal–ventral axis, accounts for dorsal enrichment of Wnt11 protein in the early *X. laevis* embryo. A seven-fold difference in the level of polyadenylated Wnt11 mRNA was reported between the dorsal and ventral halves of the embryo at the 32-/64-cell stage, against a uniform background of total mRNA for this gene (Schroeder et al., 1999); also, a 60% spike in polyA+ Wnt11 mRNA levels over the whole embryo between fertilization and the 2-cell stage was noted. This *X. laevis Wnt11* maps (using the published primers) to *X. tropicalis Wnt11b*. Our analysis shows that *Wnt11b* is strongly enriched (25.5 fold) at the vegetal pole, and this is seen in both the polyA+ and total mRNA data (Fig. 6A). Looking at the polyA+ expression levels in the individual blastomeres for Embryo 1, we note that expression in the dorsal blastomeres is slightly lower than in the ventral blastomeres, and therefore more supportive of ventral than dorsal enrichment. It appears unlikely that *Wnt11b* is significantly enriched for polyadenylated mRNAs in the dorsal hemisphere at the 8-cell stage in our data. Significant changes in polyadenylation post-fertilization have been observed for many genes (Collart et al., 2014), and *Wnt11b* is progressively deadenylated in that data, measured over the whole embryo, during the cleavage stages. There are two other Wnt11's in the *X. tropicalis* genome: one is annotated as *Wnt11*, the other is un-annotated (*H00536*), but appears to be an inverted local duplication of *Wnt11b*. This latter shows (weaker) vegetal enrichment (2.2 fold), and neither shows any evidence of differential polyadenylation. If this mechanism is operational in *X. tropicalis*, these results would suggest that the differential polyadenylation would need to take place between the 8- and 32-cell stages. We look forward to investigating this phenomenon more deeply in future work.

In the case of *Wnt11b*, we see no evidence in our data for differential polyadenylation along the dorsal–ventral axis by the 8-cell stage. Interestingly, when we extend this question to all other genes, we find no genes showing asymmetric distribution of polyadenylated mRNA along the dorsal–ventral axis (all genes have FDR>0.76). We therefore find no support in our data for differential polyadenylation along the dorsal–ventral axis, acting on a uniform distribution of underlying maternal mRNA. Neither do we find evidence (presented above) for asymmetric distribution of total mRNA along this axis, which could have allowed different levels of polyA+ mRNA to accumulate in the two hemispheres at a later stage.

Ventral to dorsal vesicle-based trafficking of Disheveled protein has been observed, after cortical rotation and before the first cleavage, and is noted to be microtubule dependent; and Disheveled protein was found to be associated with vesicle-like organelles at the vegetal pole (Miller et al., 1999). There appear to be three *Disheveled* genes in *X. tropicalis*: *Dvl1*, is expressed at very low levels, and the read counts are below our threshold for testing, whilst both *Dvl2* and *Dvl3* are much more highly expressed. Interestingly, their maternal mRNAs do not show any significant asymmetry (animal–vegetal or dorsal–ventral) in the blastomeres, in either the total mRNA or the polyA+ mRNA data (Fig. 6B). These data suggest that Disheveled protein could be produced from mRNAs more widely distributed in the egg, or oocyte, than at the vegetal pole, although we have no direct evidence for this. This would however be consistent with the role of cortical rotation to set up a dorsally directed microtubule network capable of translocating dorsalizing agents from (potentially) any part of the embryo, rather than requiring them to be localized around the vegetal pole as suggested in a recent model (Croce and McClay, 2006), although this would be inconsistent with the well established observations that material transplanted into other locations from around the vegetal pole has a dorsalizing effect (Fujisue et al., 1993, Holowacz and Elinson, 1993, Kageura, 1997).

## 4 Conclusions

Using a sensitive and stringent analysis we have found no evidence for asymmetric distribution of maternal mRNAs along either the dorsal–ventral or the left–right axes. We thus provide a considerable weight of evidence in support of the original suggestion (Flachsova et al., 2013) that the distribution of maternal mRNAs in the fertilized egg, inherited from the oocyte, is essentially undisturbed by cortical rotation.

We have shown that close to 7% of genes in the 8-cell stage embryo, with sufficient levels of expression to test for asymmetry, show discernible differential expression between the animal and vegetal poles, and that this enrichment is significantly more marked at the vegetal than the animal pole. Transcriptional activity in the 8-cell embryo is not sufficient to produce the observed polar enrichment (Collart et al., 2014, Yang et al., 2002), so the enrichment must arise from the initially asymmetric distribution of maternal mRNAs in the egg, inherited from the oocyte.

We have confirmed the lower enrichment at the animal pole compared to the vegetal pole, with all but 2/460 animal pole mRNAs enriched by<3-fold. This is significantly lower than the 3–10 folds previously reported (Grant et al., 2014, King et al., 2005), although these figures may be influenced by differences in experimental design.

It has recently been pointed out that (unlike vegetal pole localization) “the subcellular mechanism [in the oocyte] by which animal transcripts are localized has yet to be revealed” (Grant et al., 2014). We might speculate that the smaller fold changes at the animal pole (which are nevertheless consistent between embryos) are a consequence of mRNAs gradients in the oocyte or egg, set up as the internal structure of these large cells develops, rather than evidence of specific, animal pole directed transport mechanisms, or later functional requirement, although the significant enrichment of human disease genes in the animal pole cohort might suggest otherwise. There is clearly much still to be learned about the distribution of maternal mRNAs in the maturing oocyte and the early embryo.

A wide range of birth defects is known to be associated with incorrect specification of body plan asymmetry (Vandenberg et al., 2013), and understanding the molecular determinants that establish the correct axial asymmetries during embryogenesis will be critical. The results obtained here provide a new and more complete insight into spatial distribution and identification of maternal mRNA in the early embryo. This knowledge may help to shed light on these defects or other genetic congenital disorders.

## Figures and Tables

**Table 1:** Consistency of asymmetry measured at the single embryo level, compared to fold-changes measured in the pooled analysis. The first column is the number of individual embryos (maximum 5) for which a given gene is found to be asymmetrically distributed at the single embryo level (FDR<0.05). Then for either pole, we give the number of genes at each level of consistency, with the average and minimum fold-change measured in the pooled data in each group.

**Embryos**	**Vegetal**			**Animal**		
**Genes**	**Avg. fold**	**Min. fold**	**Genes**	**Avg. fold**	**Min. fold**
5	65	12.25	1.88	9	2.31	1.70
4	75	4.48	1.37	42	1.9	1.48
3	83	2.53	1.23	52	1.56	1.27
2	89	2.50	1.21	100	1.48	1.15
1	95	2.19	1.15	150	1.4	1.12
0	41	2.36	1.25	107	1.4	1.12

**Table 2:** List of mRNAs found asymmetrically distributed on the animal–vegetal axis in this study, where the pooled analysis asymmetry was confirmed individually in all five embryos; novel mRNAs are indicated in column 3. Fold change is measured between the mean expression level of pooled blastomere data at each end of the named axis over four embryos; the range of fold-changes found in the single embryos analysis is also given. The expression level is the mean expression at the higher end of the axis in the pooled data. Fold changes, where known, and gene symbols reported in other studies are indicated in relevant columns. Disease genes were annotated via orthologs of human disease associated genes from Omim (www.omim.org) and published work on protein complexes (Lage et al., 2008), and also taken directly from Xenbase (James-Zorn et al., 2013), see text. Previously published mRNAs are referenced as follows, column headings: (Grant et al., 2014;Flachsova et al., 2013; Cuykendall and Houston, 2010; King et al., 2005). Other: (Betley et al., 2002, Birsoy et al., 2005, Colozza and De Robertis, 2014, Freeman et al., 2008, Horvay et al., 2006, Kloc and Chan, 2007, Machado et al., 2005, Nakaya et al., 2004, Quick and Serrano, 2005, Rutenberg et al., 2002, Tarbashevich et al., 2011, Tarbashevich et al., 2007).

Gene	Pole	Status	Fold	Range	Expression level	Affy ID v1	Affy ID v2	Associated disease	Grant	Flachova	Cuykendall	King	Other
Xetro.D02339|grip2	Veg		134.11	(5) 49.21–213.15	6842.8	Xl.14891.1.S1_at	Xl2.52312.1.S1_at	Coffin–Lowry syndrome; diabetes mellitus, noninsulin-dependent; leukemia, juvenile myelomonocytic			10.57 (xgrip2)		Tarb 07
Xetro.G00091|nanos1	Veg		97.32	(5) 51.43–151.47	546.6	Xl.1145.1.S1_at	Xl2.1145.1.A1_at	Spermatogenic failure 12			16.04 (xcat2)		Betl 02
Xetro.E00365|pat	Veg		94.4	(5) 34.54–178.98	4588.5	Xl.38.1.S1_at	Xl2.38.1.S1_at				6.93 (xpat)	(xpat)	Mach 05
Xetro.A00019|	Veg	novel	59.12	(5) 22.21–186.66	128.9	–	–						
Xetro.G01160|ddx25	Veg		37.06	(5) 26.65–61.12	9146.2	Xl.670.1.S1_at	Xl2.670.1.S1_at		24.16 (ddx25)	(ddx25)	9.24 (dead^*^south)	(DEADSouth)	
Xetro.H00537|wnt11b	Veg		25.52	(5) 12.83–33.12	1858.3	Xl.1073.1.S1_at	Xl2.44504.2.S1_a_at				8.43 (wnt11)	(Xwnt11)	
Xetro.A00580|trim36	Veg		17.74	(5) 12.09–24.86	19264.8	Xl.6926.1.S1_at	Xl2.6926.1.S1_at			(trim36)	24.93 (MGC81170;trim36)		
Xetro.C00749|pcsk6	Veg		17.4	(5) 9.23–33.34	4505.4	–	Xl2.48635.1.S2_at						Birs 05
Xetro.F00403|sulf1	Veg		15.94	(5) 10.59–36.44	4357.1	Xl.20564.1.A1_at	Xl2.20564.1.S1_at						Free 08
Xetro.N00856|vegt	Veg		13.52	(5) 11.28–28.78	11644.3	Xl.1775.1.S1_at	Xl2.1775.1.S1_at		4.61 (vegt-a)	(vegt)	3.56 (vegt)	(VegT)	
Xetro.G00335|bicc1	Veg		12.91	(5) 9.39–26.43	11865.5	Xl.7094.1.S1_a_at	Xl2.7094.1.S1_a_at		17.12 ()		14.27 (–)	(xBic-C)	
Xetro.A02015|gdf1	Veg		12.36	(5) 7.58–22.08	2258.7	Xl.25780.1.S1_at	Xl2.25780.1.S2_at	Right atrial isomerism; transposition of great arteries, dextro-looped 3; conotruncal anomaly face syndrome; tetralogy of fallot	34.73 (gdf1)	(vg1)	3.77 (vg1)	(Vg1)	
Xetro.I00813|mogat1	Veg	novel	11.13	(5) 7.17–16.71	2464.3	Xl.7867.1.S1_at	Xl2.7867.1.S1_at						
Xetro.A01360|mov10	Veg	novel	10.28	(5) 7.66–30.84	14163.7	–	Xl2.52320.1.S1_at						
Xetro.E01314|otx1	Veg		10.25	(5) 7.43–27.25	19156	Xl.781.1.S1_at	Xl2.781.1.S1_at	Cutis laxa, autosomal recessive, type ia	19.84 (otx1)	(otx1)	7.18 (otx1)	(Xotx)	
Xetro.K02810|slc12a9	Veg	novel	9.91	(5) 5.17–30.20	617.3	–	Xl2Affx.115.1.S1_at						
Xetro.F00192|sybu	Veg		9.87	(5) 6.22–15.34	6385.3	–	Xl2.8441.1.S1_at						Colo 14
Xetro.I01736|thoc6	Veg	novel	7.94	(5) 4.54–12.64	3028.5	–	Xl2.11109.1.S1_at						
Xetro.A01296|slc25a22	Veg	novel	7.76	(5) 6.34–17.48	837.5	Xl.26141.1.S1_at	Xl2.24565.1.A1_at	Epileptic encephalopathy, early infantile, 3					
Xetro.J00911|spire1	Veg		7.74	(5) 5.48–10.35	23589.6	–	Xl2.7501.1.S1_at	Smith–Magenis syndrome; deafness, autosomal dominant 4a; glomerulosclerosis, focal segmental, 1; deafness, autosomal dominant 48	13.43 (spire1)				
Xetro.H00830|	Veg	novel	7.42	(5) 6.29–8.53	14434.3	Xl.7236.2.A1_at	Xl2.54089.1.S1_at						
Xetro.A00833|unnamed	Veg	novel	7.22	(5) 4.96–9.62	879.5	–	–						
Xetro.C01112|dnd1	Veg		7.16	(5) 5.35–25.55	3707.9	Xl.25471.1.A1_at	Xl2.29785.1.S2_at		3.60 (dnd1)		5.06 (dead^*^end)		Horv 06
Xetro.F01892|ctdspl	Veg		7.09	(5) 3.73–17.59	14334	Xl.18931.1.A1_at	Xl2.18931.1.S1_at		13.21 (ctdspl)		6.37 (nif)		
Xetro.A01886|rnf38	Veg		6.94	(5) 4.36–19.21	17191.6	Xl.5623.1.A1_at	Xl2.52368.1.S1_at		13.11 ()		10.05 (–)		
Xetro.F00384|rdh10	Veg		6.64	(5) 4.64–13.62	13302	Xl.24399.1.A1_at	Xl2.47730.1.S1_at				6.98 (MGC80820)		
Xetro.I00127|cnppd1	Veg		6.16	(5) 4.67–13.70	5967.5	Xl.7190.1.A1_at	Xl2.7190.1.S1_at		19.05 (cnppd1)		6.74 (MGC115028)		
Xetro.G00368|fgfr2	Veg		5.88	(5) 3.54–13.85	931.7	Xl.1182.1.S1_at	Xl2.21506.1.A1_at	Bent bone dysplasia syndrome; ladd syndrome; Antley–Bixler syndrome without genital anomalies or disordered steroidogenesis; gastric cancer, somatic; apert syndrome; scaphocephaly, maxillary retrusion, and mental retardation; Jackson–Weiss syndrome			11.36 (fgfr2)		
Xetro.C00709|rhcg	Veg	novel	5.8	(5) 3.72–7.05	1381.4	–	Xl2.31959.1.S1_at						
Xetro.B00467|cnksr2	Veg	novel	5.77	(5) 4.95–6.68	5149	–	Xl2.47631.1.S1_at						
Xetro.K05126|sox7	Veg		5.71	(5) 3.25–12.31	2974.8	Xl.1241.1.S1_at	Xl2.1241.1.S1_at		4.86 (sox7)				
Xetro.A02337|ppp1r3b	Veg		5.33	(5) 4.37–7.09	21289.8	Xl.7655.1.S1_at	Xl2.7655.1.S1_at	Muscle glycogenosis; diabetes mellitus, type 2, susceptibility to; mcardle disease; phosphorylase kinase deficiency of liver and muscle, autosomal recessive; glycogen storage disease 0, liver; ossification of the posterior longitudinal ligament of spine;	3.15 (ppp1r3b-a)		8.46 (MGC85023)		
Xetro.B00074|rragc	Veg	novel	5.26	(5) 4.56–8.78	7477.7	–	Xl2.7005.1.S1_at						
Xetro.A02205|acsbg2	Veg		5.06	(5) 4.18–9.59	15304	Xl.5085.1.A1_at	Xl2.5125.1.S2_at		7.41 (acsbg2)				
Xetro.I00550|raph1	Veg	novel	4.99	(5) 3.16–10.12	3402.8	Xl.15448.2.A1_at	Xl2.15448.1.S1_at						
Xetro.D02536|	Veg	novel	4.59	(5) 3.04–13.56	906.3	–	Xl2.29748.1.A1_at	Robinow syndrome, autosomal dominant; exudative vitreoretinopathy 2, x-linked; exudative vitreoretinopathy 4; [bone mineral density variability 1]; exudative vitreoretinopathy 1; parkinson disease 6, early onset;					
Xetro.D01936|plk3	Veg	novel	4.48	(5) 3.56–5.49	27440.1	Xl.19981.1.S1_at	Xl2.8085.1.S1_at	Esophageal squamous cell carcinoma, somatic; lung cancer, somatic; choroid plexus papilloma; squamous cell carcinoma, head and neck, somatic; li-fraumeni syndrome;					
Xetro.F01128|velo1	Veg		4.33	(5) 3.84–9.66	18561.9	Xl.491.1.S1_at	Xl2.491.1.S1_a_at		5.44 (velo1)			(Xvelo)	
Xetro.B00910|cldn4	Veg	novel	4.27	(5) 2.20–7.08	9651.6	Xl.6291.1.A1_at	Xl2.53796.2.A1_at						
Xetro.H00419|	Veg	novel	4.02	(5) 3.04–7.02	1090.5	Xl.690.1.S2_at	Xl2.690.1.S1_at						
Xetro.A02978|acsl1	Veg		3.87	(5) 2.30–5.18	6049.8	Xl.15591.1.S1_at	Xl2.7122.1.S1_at	Myopathy due to cpt ii deficiency; cpt deficiency, hepatic, type ia; cpt deficiency, hepatic, type ii; cpt ii deficiency, lethal neonatal; acyl-coa dehydrogenase, medium chain, deficiency of; nephronophthisis 3; peroxisomal acyl-coa oxidase deficiency;	6.33 ()		20.58 (facl2)	(XFACS)	
Xetro.B01314|rnf41	Veg	novel	3.85	(5) 2.34–6.42	6800.8	Xl.3157.1.A1_at	Xl2.3157.1.S1_at						
Xetro.D01271|pbx1	Veg	novel	3.79	(5) 2.79–4.73	2182.2	Xl.9563.1.S1_at	Xl2.40234.1.S1_at	Coloboma of optic nerve; ectopia pupillae; foveal hypoplasia 1; keratitis; optic nerve hypoplasia; aniridia; coloboma, ocular; peters anomaly;					
Xetro.D00562|pc.1	Veg		3.76	(5) 2.82–5.75	2613.3	Xl.16639.1.A1_at	Xl2.16639.1.S2_at	Pyruvate carboxylase deficiency; glutaricaciduria, type i; cardiomyopathy, familial hypertrophic 6; lacticacidemia due to pdx1 deficiency; cholesteryl ester transfer protein deficiency; pyruvate kinase deficiency; charcot-marie-tooth disease, type 2d	7.97 (pc.1)				
Xetro.G00513|sufu	Veg	novel	3.57	(5) 2.63–7.52	3813.4	Xl.10858.1.A1_at	Xl2.47662.1.S1_at	Meningioma, familial, susceptibility to; basal cell nevus syndrome; peroxisome biogenesis disorder 1b (nald/ird); peroxisome biogenesis disorder 3b; peroxisome biogenesis disorder 2b; peroxisome biogenesis disorder 1a (zellweger); medulloblastoma					
Xetro.B01376|lrp1	Veg		3.47	(5) 2.49–8.30	11835.5	Xl.10926.1.A1_at	Xl2.18251.1.S1_at	Neutropenia, cyclic; corneal dystrophy, groenouw type i; corneal dystrophy, lattice type i; corneal dystrophy, avellino type; corneal dystrophy, Reis–Bucklers type; corneal dystrophy, lattice type iiia; Bernard–Soulier syndrome, type b	9.03 (lrp1)		7.45 (–)		
Xetro.K00842|	Veg		3.32	(5) 2.59–6.49	11744.4	Xl.7672.1.S1_at	Xl2.7672.1.S1_at		7.36 ()		6.26 (–)		
Xetro.K02822|znf484	Veg	novel	3.17	(5) 2.28–3.95	484.7	–	–						
Xetro.K01814|	Veg	novel	2.92	(5) 2.31–3.36	1665.6	–	–						
Xetro.D02087|	Veg		2.92	(5) 2.68–3.81	14753.6	–	Xl2.25589.1.A1_at		3.43 (rpap3)				
Xetro.B01421|unnamed	Veg	novel	2.78	(5) 2.05–3.51	6624.5	Xl.5306.1.S1_at	Xl2.55308.1.A1_x_at						
Xetro.H01623|daam1	Veg		2.75	(5) 1.92–5.33	8593.7	Xl.25142.1.A1_at	Xl2.14901.1.A1_at						Naka 04
Xetro.D00538|fam65a	Veg		2.62	(5) 2.01–3.25	12257	Xl.11436.1.A1_at	Xl2.54196.1.S1_at				8.43 (–)		
Xetro.D01911|c1orf190	Veg		2.52	(5) 2.04–3.49	2837.4	Xl.18636.2.A1_at	Xl2.18636.2.A1_at	Cerebral dysgenesis, neuropathy, ichthyosis, and palmoplantar keratoderma syndrome	5.33 ()		10.10 (–)		
Xetro.E00863|dynlt1	Veg	novel	2.5	(5) 1.89–4.61	1843.3	Xl.25778.2.A1_at	Xl2.25778.1.S1_at						
Xetro.K00790|unnamed	Veg	novel	2.45	(5) 2.09–2.83	3740.6	–	–						
Xetro.D02050|ralgps2	Veg		2.43	(5) 1.94–3.46	8900.2	Xl.15398.1.A1_at	Xl2.15398.1.S1_at		10.21 ()		6.69 (–)		
Xetro.A00139|gpbp1	Veg	novel	2.4	(5) 2.14–5.49	1751	Xl.10420.1.A1_at	Xl2.10420.1.S1_at						
Xetro.A02066|tbc1d1	Veg	novel	2.4	(5) 1.92–2.95	10331.8	–	Xl2.51321.2.A1_at	Renal cell carcinoma, papillary; craniofacial-skeletal-dermatologic dysplasia; hypereosinophilic syndrome, idiopathic, resistant to imatinib; growth retardation with deafness and mental retardation due to igf1 deficiency; crouzon syndrome					
Xetro.A00748|slc15a4	Veg	novel	2.28	(5) 1.83–3.12	5433	Xl.11559.2.A1_at	Xl2.11559.1.S1_at						
Xetro.F01187|trak1	Veg		2.2	(5) 1.78–2.65	4548.3	Xl.3942.1.A1_at	Xl2.14609.1.A1_at		7.25 (trak1)		6.33 (–)		
Xetro.C01467|pdlim7	Veg	novel	2.09	(5) 1.60–3.61	3072.3	Xl.24906.1.S1_at	Xl2.24906.1.S1_at	Arthrogryposis multiplex congenita, distal, type 1; cap myopathy 2; cirrhosis, cryptogenic					
Xetro.B00109|eif2c1	Veg	novel	1.99	(5) 1.54–2.76	2626.4	–	–						
Xetro.D02103|kifc3	Veg	novel	1.93	(5) 1.65–2.55	2547.1	–	Xl2.47072.1.S1_at						
Xetro.E00664|	Veg	novel	1.88	(5) 1.70–2.05	5609	Xl.7576.1.A1_at	Xl2.7576.1.S1_at						
Xetro.D01799|slc35d1	Anim	novel	3.83	(5) 2.96–5.35	1912.7	Xl.15597.1.A1_at	Xl2.15597.1.S1_at	Schneckenbecken dysplasia					
Xetro.A01057|smtn	Anim		2.83	(5) 2.43–3.25	14249.3	Xl.7657.1.S1_at	Xl2.30815.1.S1_at	Prostate cancer, somatic; glioma susceptibility 1; persistent mullerian duct syndrome, type ii; parkinson disease, susceptibility to; exudative vitreoretinopathy 1; brachydactyly, type a2; parkinson disease 6, early onset; endometrial cancer, familial	4.45 (smtn)				
Xetro.C00309|slc18a2	Anim	novel	2.65	(5) 1.94–3.09	5710.6	Xl.2817.1.S1_at	Xl2.12817.1.A1_at						
Xetro.I00501|nbeal1	Anim	novel	2.38	(5) 1.79–2.89	8503.6	–	–						
Xetro.B00228|stk40	Anim	novel	2.11	(5) 1.71–2.34	7574.6	Xl.15814.3.A1_at	Xl2.29416.1.S1_at						
Xetro.I01183|nomo3	Anim	novel	1.82	(5) 1.45–2.10	6120.2	Xl.9133.1.A1_at	Xl2.56596.1.S1_at						
Xetro.E01463|bub1	Anim	novel	1.75	(5) 1.45–2.06	9400.8	Xl.754.1.S1_at	Xl2.754.1.S2_a_at						
Xetro.A01908|ptpn9	Anim	novel	1.74	(5) 1.43–1.95	3182.6	Xl.12128.1.S1_at	Xl2.883.2.S1_a_at						
Xetro.H00897|rbmx	Anim	novel	1.7	(5) 1.22–2.12	2392.2	Xl.25221.1.S1_at	Xl2.25221.2.S1_a_at	Cornelia de lange syndrome 2; oculopharyngeal muscular dystrophy; cerebral hemorrhage with amyloidosis, hereditary, dutch type; griscelli syndrome, type 1; elejalde disease; griscelli syndrome, type 3; keratosis palmoplantaris striata i, ad					

**Table 3:** Penetrance of phenotype in Slc35d1-MO injected embryos. Number of observed short-axis phenotypes in each group of embryos injected with different levels of the Slc35d1 Morpholino at the single cell stage. Observations made at Stage 32/33.

**Slc35d1-MO concentration (ng/embryo)**	**Embryos injected**	**Short-axis phenotype**	**Penetrance (%)**
10	84	81	96
20	88	86	98
30	94	88	94
40	97	94	97
