# Supplementary material for: Molecular asymmetry in the 8-cell stage Xenopus tropicalis embryo described by single blastomere transcript sequencing
Source: Dev Biol. 2015 Dec 15;408(2):252–68. doi: 10.1016/j.ydbio.2015.06.010 (PMC4684228; doi:10.1016/j.ydbio.2015.06.010)
Supplement: Supplementary file 1 — Supplementary material [file mmc1.doc]

**SUPPLEMENTARY FILES:**

Supplementary File 1: *Xenopus laevis* Affymetrix IDS to *Xenopus tropicalis* transcript

conversion.

<http://genomics.nimr.mrc.ac.uk/publications/2015-03-25/affymetrix-annotations-31jul14-Xl-to-Xt.txt>

Supplementary File 2: RNA-seq read counts for 8-cell stage embryo blastomeres.

<http://genomics.nimr.mrc.ac.uk/publications/2015-03-25/blastomere-cell-expression-data-25mar15-v2.txt>

Supplementary File 3: Genes enriched in animal or vegetal blastomeres in the 8-cell stage

embryo.

<http://genomics.nimr.mrc.ac.uk/publications/2015-03-25/asymmetrically-expressed-genes-ALL.xlsx>

Supplementary File 4: *Xenopus tropicalis* human disease orthologs

<http://genomics.nimr.mrc.ac.uk/publications/2015-03-25/Xenopus-tropicalis-diseasegenes-v2.xlsx>
